# Oral Iron Prophylaxis in Pregnancy: Not Too Little and Not Too Much!

**DOI:** 10.1155/2012/514345

**Published:** 2012-07-24

**Authors:** Nils Milman

**Affiliations:** Department of Obstetrics, Næstved Hospital, DK-4700 Næstved, Denmark

## Abstract

An adequate supply of iron is essential for normal development of the fetus and newborn child. Iron deficiency and iron deficiency anemia (IDA) during pregnancy increase the risk of preterm birth and low birth weight. Iron is important for development of the fetal brain and cognitive abilities of the newborn. Children born to iron-deficient mothers will start their lives suffering from iron deficiency or even IDA. Oral iron prophylaxis to pregnant women improves iron status and prevents development of IDA. The Danish National Board of Health has since 1992 recommended prophylactic oral iron supplements to all pregnant women and the currently advocated dose is 40–50 mg ferrous iron taken between meals from 10 weeks gestation to delivery. However, 30–40 mg ferrous iron is probably an adequate dose in most affluent societies. In developed countries, individual iron prophylaxis guided by iron status (serum ferritin) has physiological advantages compared to general iron prophylaxis. In contrast, in most developing countries, general iron prophylaxis is indicated, and higher doses of oral iron, for example, 60 mg ferrous iron or even more should be recommended, according to the present iron status situation in the specific populations of women of fertile age and pregnant women.

## 1. Introduction

In a global perspective, the most frequent nutritional insufficiency is definitely iron deficiency, which is encountered with a high prevalence in women of fertile age as well as in pregnant and postpartum women [1]. In many developing countries, iron deficiency anemia (IDA) in pregnancy is more the rule than the exception with a prevalence of approximately 52% [2]. In the prosperous western societies, the frequency of IDA is lower due to better nutrition, approximately 25% in pregnant women not taking iron supplements and less than 5% in women taking prophylactic iron supplements of 40–60 mg ferrous iron per day [3, 4]. The World Health Organization (WHO) estimates that the number of anemic pregnant women in the world is ~56 million and the majority of these women (75–80%) have IDA [2]. Of these women, ~7 million are residents in Europe and the Americas and the remaining 49 million in more or less developed countries. In Europe, the number of anemic pregnant women is ~2.5 million.

An adequate body iron status is, among other factors, a prerequisite for a normal and healthy gestation, a normal development of the fetus and a healthy newborn baby. Iron deficiency, even without IDA, reduces the cognitive abilities and physical performance in nonpregnant women [5, 6]. In pregnant women, IDA is associated with preterm delivery, low birth weight of the newborns [7] as well as iron deficiency in the newborns.

Furthermore, untreated iron deficiency and IDA in the third trimester strongly predisposes to postpartum iron deficiency and IDA [8], which are associated with decreased physical abilities and psychic disturbances including emotional instability, depression, stress, and reduced cognitive performance tests [9, 10].

### 1.1. Dietary Iron Intake Is Inadequate in the Majority of Pregnant Women

**Figure 1:**
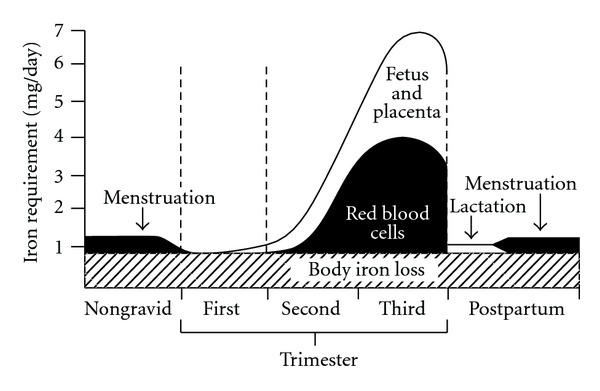
Requirements for absorbed iron in pregnant and lactating women; reproduced with permission [13].

In gestation, the total demands for absorbed iron are approximately 1240 mg (Table 1). The demand for absorbed iron increase steadily during pregnancy from 0.8 mg/day in the initial 10 weeks of gestation to 7.5 mg/day in the last 10 weeks of gestation as shown in Figure 1. During the entire gestation period, the average demand for absorbed iron is 4.4 mg/day [11–13].

Danish women of fertile age have a mean dietary iron intake of 9 mg/day, that is, the majority of the women (more than 90%) have an iron intake which is definitely below the recommended intake of 15–18 mg/day in women of fertile age [14].

In general, women do not make substantial changes in their dietary habits when they become pregnant. In a Norwegian dietary survey, more than 800 women were examined prior to pregnancy as well as in 17 and 33 weeks of gestation. Energy intake and the composition of the diet were similar before and during gestation. Mean energy intake was 8.9 MJ/day at the dietary assessments [15], which corresponds to the energy intake in nonpregnant Danish women. The average distributions of energy derived from protein, fat and carbohydrates were identical, 14, 36, and 50%, respectively. Mean dietary iron intake was 11 mg/day and below 18 mg/day in 96% of the women [15]. Pregnant women in the UK have a mean dietary iron intake of 10 mg/day [16] and pregnant Bavarian women a mean dietary iron intake of 13 mg/day [17], that is, far below the intake of 27–30 mg/day, which is recommended in Germany and USA.

Body iron balance and iron status are influenced by the magnitude of dietary iron intake in combination with the bioavailability of dietary iron. Generally, dietary iron intake is proportional with the energy intake. In the developing countries as well as in the western countries, almost all women have a dietary iron intake, which is inadequate to fulfill the body iron demands in the second and third trimester of pregnancy. The low dietary iron intake is partly due to a low intake of meat, poultry, and fish products and partly due to a low energy intake elicited by the sedentary lifestyle, which has become dominant in many western and some developing countries.

From a nutritional point of view, food iron exists in two main forms, heme iron and nonheme iron. The major part of dietary iron consists of nonheme iron. However, heme iron has a higher bioavailability than nonheme iron, it is more easily absorbed and therefore plays a major role in maintaining a favorable body iron status. Iron absorption is increased by consumption of food items with a high content of iron with a high bioavailability, for example, beef, pork, poultry, fish, and food items containing blood products. Besides heme iron, meat contains a promoter of nonheme iron absorption, the so-called “meat factor”. Calf and pork liver have a high content of iron with a good bioavailability, but the Danish Health Authorities advise pregnant women not to consume pork liver and pork liver paté due to the high content of vitamin A, which possibly may cause malformations in the fetus [18]. In regions of the world where the diet is predominantly vegetarian and there is a high consumption of tea, for example, South East Asia, iron status in pregnant women is lower than in regions with a low intake of tea and a higher consumption of meat products (e.g., European countries) [2].

The absorption of iron is inhibited by calcium, which is most abundant in milk and milk products, by polyphenols in tea, coffee, and some wines and by phytates in cereal products, for example, bread. Even under the most favorable conditions, only 30% of dietary iron can be absorbed, corresponding to 3 mg iron/day with an iron intake of 9-10 mg/day, that is, considerably below the average daily iron requirements during pregnancy. In the average Danish diet the bioavailability of iron is approximately 18%. A higher dietary iron intake with a higher bioavailability would imply fundamental changes in nutritional patterns, and it is not realistic to assume that such changes can be implemented in pregnant women. In the Nordic countries, the Nordic Council of Ministers elaborates the common “Nordic Nutrition Recommendations”. As a consequence of the fact that dietary iron content is inadequate to fulfil the need for iron in the majority of pregnant women, the Nordic guidelines refrain from giving exact recommendations for dietary iron intake during pregnancy [19]. As mentioned above some countries advocate a dietary iron intake of 27–30 mg/day in pregnancy.

## 2. Iron Prophylaxis in Pregnancy: A Confusing Situation

There is no consensus in the developed Western countries concerning iron prophylaxis to pregnant women. In fact, each country has their separate recommendation on this issue. Some countries (e.g., Denmark) advocate iron prophylaxis, while others (e.g., UK, Norway) do not. Some countries (e.g., Germany) have not yet established national guidelines. The European Union in 1993 concluded that “the physiologic solution for covering the high iron requirements in pregnancy is to use iron from stores. The problem, however, is that very few women, if any, have sufficient iron stores of this magnitude, greater than 500 mg. Therefore, daily iron supplements are recommended in the latter half of pregnancy”. The European Union sponsors the European Micronutrient Recommendations Aligned (EURRECA) with the intensions of harmonising nutrient recommendations across Europe with special focus on vulnerable groups, including pregnant women. The Nordic Nutrition Recommendations in 2004 stated that “an adequate iron balance during pregnancy demands body iron stores of at least 500 mg. The physiological need for iron in the second half of gestation cannot be covered by dietary intake of iron” [19].

### 2.1. Iron Is Important for Fetal Development

Iron is essential for a normal development of the fetus, and it is therefore crucial to prevent and avoid iron deficiency during the entire gestation period. A physiological and logical way to obtain this goal is to prevent (or treat if indicated) iron deficiency in the pregnant woman.

The fetus uses the major part of its iron supply to the synthesis of hemoglobin, but iron also plays an important role in the development of several vital organ systems, including the central nervous system where iron containing enzymes are involved in many metabolic processes. The growing brain has a demand for a balanced supply of iron across the blood-brain barrier [20]. In the fetus and newborn babies, iron deficiency may cause permanent damage to the brain, which negatively affects the intelligence, cognitive abilities and behavior during growth and later in life [21].

### 2.2. Iron Status in the Newborn

To a large extent, the newborn's iron status depends on the woman's iron status during pregnancy. Infants born to mothers who have taken iron supplements during gestation have larger body iron reserves (serum ferritin) than infants born to mothers who have taken placebo [3, 22].

 Therefore, infants born to iron supplemented mothers have a smaller risk of developing iron deficiency and IDA in the first years of life [23]. Another factor, which is of importance for the newborn's iron status is the volume of blood, which is transferred from the placenta before the umbilical cord is clamped. In full-term neonates, delayed clamping for a minimum of two minutes following birth is beneficial for hematological status and iron status [24]. It increases the newborn's blood volume by approximately 30% and decreases the risk of iron deficiency during infancy [25].

### 2.3. Birth Weight Is Influenced by the Mothers Iron Status

Experiences from both developing and developed countries show that IDA in pregnant women increases the risk of preterm birth and low birth weight of the newborn [7, 26–28]. Pregnant Nepal-women, who took daily supplements of 60 mg ferrous iron and 0.4 mg folic acid from 11 weeks gestation gave birth to children with markedly higher birth weight than did non-supplemented women [28]. A study from USA in low-income women showed that IDA during pregnancy doubled the risk of preterm birth and tripled the risk of having a baby with low birth weight; a daily supplement of 65 mg ferrous iron reduced the frequency of preterm birth and low birth weight [7]. In another study, a daily supplement of 30 mg ferrous iron started before 20 weeks gestation induced higher birth weight of the newborn compared to non-supplemented women. These studies also point to the fact that iron supplements should be started in early pregnancy in order to obtain the best effects on the mother's course of gestation, on the development of the fetus and on the newborns birth weight.

## 3. Iron Supplements in Pregnancy—How Little is Enough?

In healthy women, serum ferritin is a reliable biomarker for mobilizable body iron reserves, that is, iron status. A ferritin concentration below 15–20 *μ*g/L indicates the presence of iron depletion and iron deficiency. When in addition there is low hemoglobin, the criteria for IDA are substantial. Many studies have shown that pregnant women taking iron supplements have higher iron status and higher hemoglobin compared to women not taking supplements [3]. The differences in iron status are recognizable many months after the women have given childbirth [2]. Pregnant women who do not take iron supplements often present with iron deficiency and IDA and in European countries IDA is more frequent among immigrants from the Middle and Far East [29] than in ethnic Europeans.

In Scandinavia approximately 40% of nonpregnant women in the fertile age have a low iron status (i.e., serum ferritin <30 *μ*g/L) and 4% have (unrecognized) IDA [14]. Among healthy ethnic Danish pregnant women, not taking iron supplements, 50% developed iron deficiency and 21% IDA, whereas among women taking 66 mg ferrous iron daily from 14 weeks gestation, 10% developed iron deficiency in late pregnancy but none displayed IDA [3].

Previously, the recommended doses of prophylactic iron supplements in pregnancy were quite high, about 100–200 mg ferrous iron daily [22]. Considering the potential side effects of iron it is important to define the smallest dose of iron, which is effective to fulfill the anticipated goals. A study of Danish pregnant women evaluated the effect of 20, 40, 60, and 80 mg ferrous iron daily from 18 weeks gestation to delivery. It appeared that a dose of 20 mg ferrous iron was inadequate to prevent iron deficiency in a substantial number of women. However, 40 mg ferrous iron prevented IDA in more than 95% of the women. Furthermore, there were no significant differences in iron status between women taking 40, 60, and 80 mg iron (Table 2) [4]. A study has compared the effect of prophylactic oral iron (ferrous sulphate 80 mg/day from ~22 weeks gestation) with prophylactic intravenous iron in repeated doses of 200 mg (iron sucrose total dose 400 or 600 mg). There was no clinically significant difference in hematological, maternal, and fetal outcomes in the oral iron group compared with the intravenous iron group. However, women taking oral iron had lower serum ferritin prior to delivery than women having 600 mg intravenous iron [30].

Could a daily multivitamin-multimineral supplement especially designed for pregnant women, which in Denmark contains 18–27 mg ferrous iron be used for iron prophylaxis? Unfortunately not, one study has shown that 72% of pregnant women taking a multivitamin-multimineral supplement containing 18 mg ferrous iron develop iron deficiency [31]. The absorption of iron from these tablets has not been adequately investigated but is probably low due to the absorptive interaction of iron with the other divalent metal ions contained in the tablets (zinc, copper, manganese, selenium, chromium, molybdenum, and sometimes calcium). Therefore it appears rational to administer iron supplements in separate tablets, which only contains iron.

During pregnancy, the oxidative stress increases to reach maximum levels at 14–24 weeks gestation [32, 33]. However, in an animal model, IDA *per se* was shown to increase oxidative stress levels in the organs and in placenta as well as hypoxia and inflammation in placenta [34]. However, daily iron supplements may also contribute to an increase in oxidative stress [35] and may induce damage to the intestinal epithelium due to high local concentrations of iron-generated free radicals.

Another concern associated with oral ferrous iron is the possible increase in the plasma concentration of the highly reactive nontransferrin bound iron. The increase in nontransferrin bound iron appears to be related to the iron dose and is most pronounced at high doses [36]. For these reasons the recommended dose of iron should be the smallest to be effective and should preferably be administered in a slow-release formula.

The daily diet contains a number of substances (e.g., calcium, polyphenols, phytates) that inhibit the absorption of iron by approximately 40% [37]. Consequently, ferrous iron supplements should be taken between meals, preferably with fruit juice containing vitamin C [38], which enhances absorption, whereas milk, coffee, and tea inhibit absorption.

## 4. Side Effects of Oral Iron Supplements

Among women it is a widespread opinion that ferrous iron tablets cause gastrointestinal discomfort. Gastrointestinal side-effects are dose dependent and usually encountered at ferrous iron doses above 100 mg/day [39]. However, controlled studies have shown that the frequencies of gastrointestinal symptoms in pregnant women taking 105 mg ferrous iron daily are not significantly different from women taking placebo [40]. Another study found no difference in gastrointestinal symptoms in pregnant women taking 80 versus 20 mg ferrous iron daily except a slightly higher frequency of constipation at the higher dose [4, 41].

Most ferrous iron formulas contain ferrous sulphate or ferrous fumarate. The iron chelate ferrous bisglycinate (Ferrochel) appears to be very well absorbed [42] and to have fewer gastrointestinal side effects than conventional ferrous iron salts. Also the oral ferric iron polymaltose complex (Maltofer) offers at least equivalent efficacy and a superior safety profile compared to ferrous sulfate for treatment of IDA during pregnancy [43]. Consequently, the anxiety for potential gastrointestinal discomfort does not seem to be justified as an argument against low-dose prophylactic iron supplementation to pregnant women.

### 4.1. Iron Prophylaxis: General or Individual?

In developing countries with sparse health resources, iron prophylaxis in pregnancy should appropriately be recommended as a general prophylaxis given to all women. In developed counties, where ample health resources are available, the question of iron prophylaxis should focus on the advantages/disadvantages of general versus individual prophylaxis. General iron prophylaxis means that all pregnant women are recommended to take iron supplements irrespective of their iron status. Individual prophylaxis indicates that iron supplements are adjusted according to the woman's iron status. From a nutritional and physiological point of view, individual iron prophylaxis is preferable to general iron prophylaxis. Iron supplements may possibly decrease the absorption of other essential divalent metal ions, for example, zinc [44] and increase the oxidative stress both locally in the intestines and generally in the body [35, 45]. Ideally, the prophylactic iron dose should therefore be the lowest possible dose, which is sufficient to prevent iron deficiency and IDA, and this purpose is most adequately obtained by individual prophylaxis.

Iron status can be defined according to the serum ferritin concentration in otherwise healthy women without ongoing inflammation. By analysis of serum ferritin either shortly before pregnancy or in early pregnancy, it is possible to categorize women in three groups: (a) those with low iron status (ferritin <30 *μ*g/L) who either already have or are in overt risk of developing iron deficiency and IDA; (b) those with intermediate iron status (ferritin 30–70 *μ*g/L) and moderate risk of iron deficiency and IDA; (c) those with adequate iron status (ferritin >70–80 *μ*g/L) with minimal or no risk of iron deficiency. Healthy pregnant women having ferritin above 70–80 *μ*g/L appear to be in safe water concerning iron deficiency as their body iron reserves are 500 mg or more, which is adequate to complete a pregnancy without taking iron supplements [19].

Genetic hemochromatosis is a group of disorders/diseases, which are characterized by excessive body iron overload [46, 47]. In populations of northern European ancestry (Iceland, Norway, Sweden, Denmark, United Kingdom, Ireland, northern France, northern Germany) mutations on the *HFE* gene, which cause genetic hemochromatosis type 1 is the most frequent hereditary disorder with a recessive inheritance. In Denmark, 0.4% of the population is homozygotes and 11% are heterozygotes [46]. Homozygous and the majority of heterozygous women will certainly not benefit from iron supplements in a general supplementation program, on the contrary this may increase body iron load and aggravate their disorder.

## 5. General Iron Prophylaxis

In Denmark, the National Board of Health has since 1992 recommended general iron prophylaxis to pregnant women with 50–70 mg ferrous iron daily from 20 weeks gestation. This recommendation was based on the outcome of a Danish study of iron supplementation during pregnancy, which demonstrated that 66 mg of ferrous iron as fumarate taken between meals was capable of preventing iron deficiency, and IDA [3]. In 2008, according to the results of a subsequent Danish dose response study [4], the guidelines were adjusted towards a lower dose of 40–50 mg ferrous iron daily from 10 weeks gestation until delivery. It is advocated that the iron supplement should be taken separately between meals in order to ensure an optimum absorption. Danish standard multivitamin-multimineral tablets contain 10–14 mg ferrous iron per tablet, while those designed for pregnant women contain 18–27 mg iron. This iron is not included in the calculation of the total iron intake, because it probably is very poorly absorbed (see above) [31], due to competition with the other minerals [44] and components contained in multivitamin-multimineral tablets.

As a guideline, pregnant women in developed countries should be recommended 30–40 mg ferrous iron daily during pregnancy. Iron tablets designed specifically for pregnant women (GraviJern, Ferrosan—a part of Pfizer Inc., Denmark) contain 40 mg ferrous iron as fumarate in a slow release formulation.

In many developing countries, the prevalence of low iron status, iron deficiency, and IDA in women of fertile age and in pregnant women is considerably higher than in the Western countries [2] and consequently the prophylactic iron dose should be higher. The WHO guidelines have recommended that iron is provided to pregnant women during antenatal visits for daily supplementation of 60 mg elemental iron, for 6 months during pregnancy and three months postpartum [48]. Iron supplements of 60 mg ferrous iron daily appear sufficient to produce maximal hemoglobin response [49, 50] and appear to be adequate to prevent IDA in pregnant Danish women [3, 4] as shown in Table 2.

## 6. Individual Iron Prophylaxis

The serum ferritin concentration should be analyzed when pregnancy is planned or as early in pregnancy as possible, preferably within 10 weeks gestation. Each *μ*g/L of ferritin indicates approximate mobilizable body iron reserves of 7–7.5 mg [51]. Multivitamin-multimineral tablets (preferably without iron) containing 0.4 mg folic acid should be recommended from the time pregnancy is planned.

1. Ferritin above 70–80 *μ*g/L (~20–25% of pregnant Danish women): body iron reserves are larger than 500 mg and iron supplements are not indicated. If ferritin is above 100–150 *μ*g/L, consider to check for inflammation, kidney disease, liver disease, hereditary hemochromatosis, cancer.
2. Ferritin in the range of 30–70 *μ*g/L (~40% of women): iron reserves are 200–500 mg. Advocated iron supplements are 30–40 mg ferrous iron daily.
3. Ferritin below 30 *μ*g/L (~40% of women): iron reserves are small and depleted in those women having values below 15 *μ*g/L: advocated iron supplements are 60–80 mg ferrous iron daily.

Individual iron prophylaxis has since 2002 been advocated by the Danish National Food Institute and since 2005 by the Danish Council of Nutrition. Both institutions conclude that iron supplements should be restricted to women with a clear demand for extra iron [11]. However, the Danish National Board of Health, having the final decisive authority, still needs to approve these recommendations. In Sweden, individual iron prophylaxis has been recommended by the Swedish Society for Obstetrics and Gynaecology since 2008 [52].

### 6.1. When Should the Women Start on Iron Supplements?

Earlier recommendations advocated that the best time to start on prophylactic iron was at 20 weeks gestation. This limit was chosen according to the results of studies showing increasing iron absorption after 20 weeks gestation [53–55]. However, analysis of these reports shows that the majority of the examined women with increased iron absorption had marked iron deficiency! Pregnant women with adequate iron reserves display a nearly “normal” iron absorption throughout pregnancy [53, 54]. Considering the significance of iron for the fetal brain development, course of pregnancy and birth weight of the newborn, low-dose iron prophylaxis should probably start when pregnancy is planned or as early in pregnancy as possible. In most countries recommending iron prophylaxis, this is initiated at the first visit to the antenatal care clinic, which may vary from 10 to 20 weeks gestation according to the structure of antenatal care in various countries. The Danish National Board of Health has recently changed their recommendation for starting iron prophylaxis from 20 to 10 weeks gestation.

## 7. What about Iron Supplements in the Lactation Period?

There are no prospective controlled studies concerning iron supplements postpartum. Therefore, in the lactation period, empirical solutions should be activated. In developing countries the WHO guidelines recommend that iron supplementation should be continued 12 weeks after partum [48]. If the woman has taken iron prophylaxis during pregnancy and presents an adequate iron status prior to childbirth and has small or normal blood losses at delivery, iron supplementation is hardly indicated in the lactation period. However, if the woman suffers peripartum blood losses greater than 400–500 mL or presents with acute bleeding anemia after delivery, her iron status should be checked and if low, oral iron treatment with 100 mg ferrous daily should be started and continued for at least 12 weeks after hemoglobin has increased to normal level [56].

If iron deficiency or IDA is present prior to delivery, it will definitely be aggravated postpartum due to blood losses. Therefore, high-dose oral iron treatment is indicated for a prolonged period, and intravenous iron therapy should be considered in women who do not respond adequately to oral iron within a couple of weeks [56].

## 8. Conclusion

In developing countries, a large fraction of women of fertile age have iron deficiency and IDA. Even in developed countries, this problem is substantial. Pregnancy induces extraordinary high demands for iron in order to increase maternal red blood cell mass and secure a normal development of the fetus. The iron demands cannot be fulfilled solely by dietary iron intake. Therefore, low-dose oral iron supplementation is indicated in the majority of pregnant women (e.g., 60 mg ferrous iron in developing and 30–40 mg in developed countries). In developing countries, general iron prophylaxis is most feasible, while in developed countries, individual iron prophylaxis should be considered. Prophylactic programs should be structured according to the iron status of women of fertile age in the specific regions/countries. In developed countries, even though there exits substantial evidence of the positive effects of iron supplements on so-called “soft values”, that is, iron status and hematological status, there is only a small or no effect on the “hard values”, that is, maternal and fetal outcome of pregnancy and delivery [50, 57]. This situation fuels the continuing discussion between the supporters and opponents of iron supplementation. However, for the benefit of pregnant women and their children, it is important that a common consensus will be reached in the near future.

## 9. Effects of Oral Iron Supplements

*Beneficial*

 Pregnant women:
  - lower prevalence of iron deficiency and IDA,
  - improved physical and psychical well-being.
 Postpartum women:
  - higher iron status at delivery,
  - lower prevalence of postpartum iron deficiency, and IDA due to peripartum blood losses.
 Fetuses and newborns:
  - beneficial for development of the brain and other organs,
  - lower frequency of premature birth,
  - decreased prevalence of low birth weight at term and low for gestational age birth weight.
 Infants:
  - larger body iron reserves at birth,
  - lower prevalence of iron deficiency and IDA in the initial 2 years of life.

*Disadvantageous*

 Pregnant women:
  - increased oxidative stress locally in the small intestines,
  - increased oxidative stress in the body in general,
  - increase in plasma nontransferrin bound iron,
  - gastrointestinal side effects at high iron doses,
  - accelerated body iron overload in women with (nondiagnosed) genetic hemochromatosis.

## Figures and Tables

**Table 1:** Iron balance in normal pregnancy and delivery, approximate figures.

Gross iron demands	
Obligatory iron loss (0.8 mg × 290 days)	230 mg
Increase in red cell mass	450 mg
Newborn baby (weight 3500 g)	270 mg
Placenta and umbilical cord	90 mg
Blood losses at delivery	200 mg

Total gross	1240 mg

Net iron demands	

Menostasia in pregnancy	−160 mg
Postpartum decrease in red cell mass	−450 mg

Total net iron demands	630 mg

**Table 2:** Prevalence of iron deficiency and iron deficiency anemia during pregnancy in Danish women according to ferrous fumarate iron supplements taken between meals from 18 weeks gestation to delivery [4].

	Iron deficiency^∗^			Iron deficiency anemia^∗∗^		
Gestational week	18	32	39	18	32	39
*n*=	427 (%)	310 (%)	269 (%)	427 (%)	310 (%)	269 (%)
Ferrous iron (mg/day)						
20	6.1	50.0	28.8	0	1.3	10.0
40	9.0	26.0	11.1	1.9	1.3	4.5
60	6.9	16.9	10.0	0	0	0
80	10.8	13.2	9.0	0	0	1.5
*P* value^∗∗∗^	NS	<0.0001	<0.01	NS	NS	0.02

^∗^Serum ferritin <13 *μ*g/L.

^∗∗^Pregnancy: ferritin <13 *μ*g/L and hemoglobin <106 g/L (week 18) <105 g/L (week 32) <115 g/L (week 39).

^∗∗∗^NS: nonsignificant.
